# Lower Blood Lipid Level Is Associated with the Occurrence of Parkinson's Disease: A Meta-Analysis and Systematic Review

**DOI:** 10.1155/2022/9773038

**Published:** 2022-06-09

**Authors:** Xue Hong, Wenting Guo, Shanshan Li

**Affiliations:** ^1^General Medical Department, Changshou Community Healthcare Center of Putuo District, Shanghai 200060, China; ^2^General Medical Department, West Nanjing Road Community Healthcare Center of Jingan District, Shanghai 200041, China; ^3^Emergency Department, Huashan Hospital Affiliated to Fudan University, Shanghai 200040, China

## Abstract

**Background:**

The changes of blood lipid levels in patients with Parkinson's disease (PD) and its clinical relevance remain unclear. We aimed to evaluate the potential association of blood lipid and the occurrence of PD, to provide evidence to the clinical treatment and nursing care of PD.

**Methods:**

We searched PubMed, Medline, Web of Science, Cochrane Library, Wanfang Database, Weipu Database, and China National Knowledge Infrastructure for studies related to the blood lipid levels and PD until November 30, 2021. Two researchers independently screened the literature and extricated the data including the levels of total cholesterol (TC), triglycerides (TG), high-density lipoprotein cholesterol (HDL-C), and low-density lipoprotein cholesterol (LDL-C). Newcastle-Ottawa Scale (NOS) was used to evaluate the quality of included studies. RevMan5.3 and Stata 12.0 software were used for statistical processing and analysis.

**Results:**

A total of 15 cohort studies with 9740 participants involving 2032 PD patients and 7708 controls were included. Meta-analysis indicated that TC (SMD = −0.29, 95% CI −0.55∼−0.03, *P*=0.04), TG (SMD = −16.83, 95% CI −20.71∼−12.95, *P* < 0.001), HDL-C (SMD = −0.14, 95% CI −0.26∼−0.02, *P* < 0.001) and LDL-C (SMD = −0.26, 95% CI −0.50∼−0.01, *P*=0.04) level in the PD patients was significantly lower than that of health controls. Sensitivity analysis indicated that the results were stable. No significant publication bias was found between the synthesized outcomes.

**Conclusions:**

Lower blood TC, TG, HDL-C, and LDL-C level are associated with the occurrence of PD. Limited by sample size and study population, further high-quality, large-sample clinical trials in different areas are needed to further determine the relationship between blood lipids and PD in the future.

## 1. Background

Parkinson's disease (PD) is a common neurodegenerative disease, and the incidence of PD in people over 65 years old is nearly 2% [1–3]. The clinical symptoms of PD include motor symptoms and nonmotor symptoms. The motor symptoms of PD are mainly motor retardation, resting tremor, muscle rigidity, and other symptoms, while the common nonmotor symptoms are cognitive dysfunction, including PD mild cognitive impairment (PD-MCI) and PD dementia (PDD) [4, 5]. MCI is the early stage of PDD, and about 60% of PD-MCI patients will develop PDD within 5 years [6]. The occurrence and development of PD dyskinesia and cognitive dysfunction seriously affect the lives of patients and their families, so it is of great significance to prevent and delay PD dyskinesia and cognitive dysfunction in the elderly patients.

Recent studies [7–9] have shown that blood lipids and blood uric acid levels may be related to the occurrence of PD. However, the results of studies on the relationship between blood lipids and PD are currently quite different. Previous studies [10, 11] have shown that reduced levels of total cholesterol (TC), triglycerides (TG), high-density lipoprotein cholesterol (HDL-C), and low-density lipoprotein cholesterol (LDL-C) in PD patients may be a sign of the severity of PD. However, other studies [12, 13] have shown that TC may have nothing to do with the pathogenesis of PD. Therefore, this meta-analysis aims to further explore the blood lipid level of PD patients and its correlation with the occurrence and development of PD, and to provide evidence support for the treatment and care of clinical PD.

## 2. Method

We aimed to prepare and report this meta-analysis and systematic review according to the Preferred Reporting Items for Systematic Reviews and Meta- Analyses (PRISMA) [14].

### 2.1. Literature Research and Retrieval

The two authors searched PubMed, Medline, Web of Science, Cochrane Library, Wanfang Database, Weipu Database, and China National Knowledge Infrastructure CNKI) databases on all publicly published original documents related to blood lipid levels and PD up until March 5, 2022. The search strategy (Supplementary file 1) used was (“PD” OR “Parkinson disease”) AND (“serum lipid” OR “blood lipid” OR “dyslipidemia” OR “cholesterol” OR “triglyceride” OR “low-density lipoprotein cholesterol” OR “LDL-C” OR “high-density lipoprotein cholesterol” OR “HDL-C”). At the same time, we also manually search the relevant references of the included literature to reduce missed detection. If the data of the literature were not clear or there were data missing, then we attempted to obtain relevant data through multiple contact with authors to make the included literature and data as comprehensive as possible.

### 2.2. Inclusion and Exclusion Criteria

The literature inclusion criteria for this meta-analysis are as follows: (1) publicly published case-control or cohort studies on the relationship between blood lipids and PD, and data such as sample size, mean, and standard deviation could be extracted; (2) the age of the research population was ≥18 years; (3) the diagnosis of PD met the diagnostic criteria, and the control group was matched non-PD population; (4) the language of the article was limited to English and Chinese.

The literature exclusion criteria for this meta-analysis are as follows: (1) we removed reviews, case reports, meeting records, and animal and cell experiments; (2) repeated published articles; (3) reports in which data could not be extracted for statistical analysis.

Two researchers independently reviewed the title and abstract of the literature for preliminary screening, and read the full text of the literature according to the inclusion and exclusion criteria to determine whether it was selected based on the inclusion and exclusion criteria.

### 2.3. Data Extraction

The data extraction of included studies in this meta-analysis was carried out independently by two researchers. The relevant data were extracted according to the data extraction table designed in advance, and the results were cross-checked. In case of disagreement, the third researcher would be introduced to discuss and resolve the disagreement. The extracted content mainly included: first author, publication year, region, research type, sample size, observation index including the TC, TG, HDL-C, and LDL-C level.

### 2.4. Literature Quality Assessment

This meta-analysis used the Newcastle–Ottawa Scale (NOS) [15] to evaluate the quality of the included cohort studies. NOS contains a total of 8 items, which include 4 items related to the study populations (total 4 points), 1 item related to the comparability between groups (total 2 points), 3 items related to the exposure determination (total 3 points). The total score of NOS is 9 points. In this meta-analysis, two researchers independently conducted quality evaluation. If there was a disagreement, the literature quality score would be determined through discussion with the third researcher.

### 2.5. Statistical Analysis

This meta-analysis uses RevMan5.3 and Stata 12.0 software for statistical processing. We first conducted a heterogeneity test for the included studies. If there was no heterogeneity in the outcomes of included studies (*I*^2^ < 50%, *P* > 0.05), the fixed effects model would be used. Otherwise, the random effects model would be used and the possible heterogeneity will be considered by conducting subgroup analysis and regression analysis. Standard mean difference (SMD) and 95% confidence interval (95% CI) were calculated for synthesized effect analysis, and forest plot was drawn, sensitivity analysis and publication bias assessment were performed. Sensitivity analysis was used to judge the stability of the results. A funnel chart and Begg's test were used to assess publication bias. In this meta-analysis, the difference was statistically significant with *P* < 0.05.

## 3. Results

### 3.1. Study Selection

**Figure 1:**
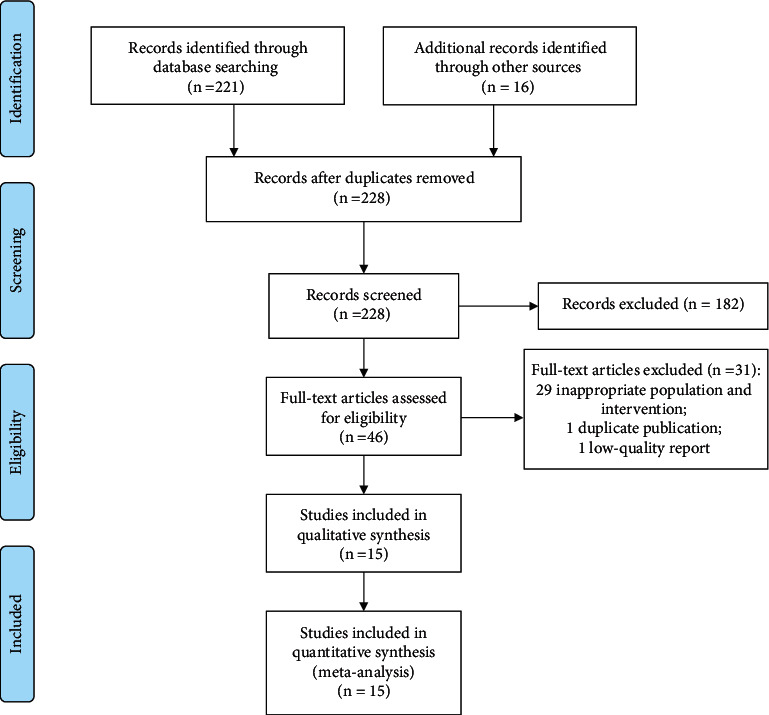
PRISMA flow diagram of study inclusion.

As shown in Figure 1, we had initially retrieved 237 documents. After excluding duplicate publications and documents that obviously did not meet the inclusion criteria, 46 full-text articles were further obtained and read, and after excluding articles with incomplete data or inappropriate population and interventions, 15 studies [16–30] were finally included for meta-analysis in this present study.

### 3.2. The Characteristics and Quality of Included Studies

Of the included 15 cohort studies [16–30], a total of 9740 participants were involved, of whom there were 2032 PD patients and 7708 controls. As shown in Table 1, these studies were published between 2007 and 2021 and were conducted in 6 countries with different geographic locations, 7 studies were conducted in Asian countries, and 8 studies were conducted in non-Asian countries. Participants included male and female patients. The highest NOS score is 8 points and the lowest is 6 points, indicating that the quality of the included studies is good.

### 3.3. Meta-Analysis

**Figure 2:**
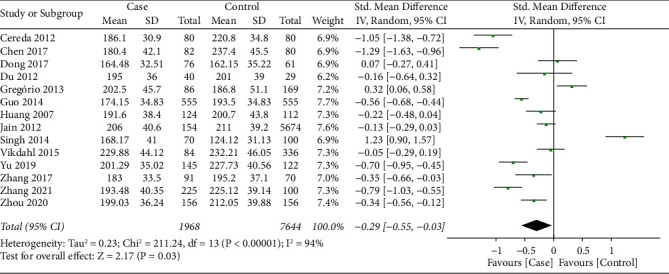
Forest plot for synthesized TC level.

14 studies [16–25, 27–30] reported the TC level between PD patients and health controls. There was significant heterogeneity on the synthesized TC level (*I*^2^ = 94%, *P* < 0.001), therefore random effect model was applied for data synthesis. As showed in Figure 2, meta-analysis indicated that TC level in the PD patients was significantly lower than that of health controls (SMD = −0.29, 95%CI −0.55∼−0.03, *P*=0.03).

**Figure 3:**
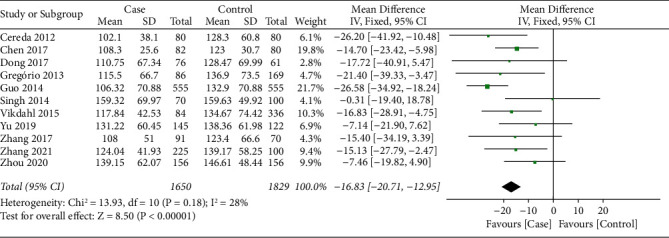
Forest plot for synthesized TG level.

11 studies [16, 17, 19–21, 24, 25, 27–30] reported the TG level between PD patients and health controls. There was no significant heterogeneity on the synthesized TG level (*I*^2^ = 28%, *P*=0.18); therefore, the fixed effect model was applied for data synthesis. As shown in Figure 3, meta-analysis indicated that TG level in the PD patients was significantly lower than that of health controls (SMD = −16.83, 95% CI −20.71∼−12.95, *P* < 0.001).

**Figure 4:**
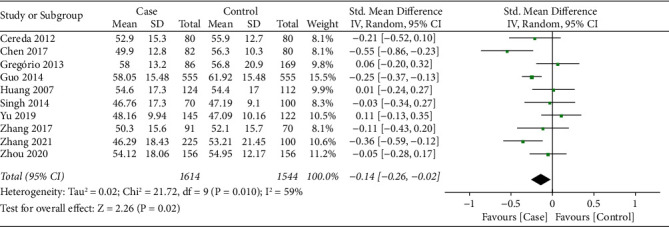
Forest plot for synthesized HDL-C level.

10 studies [16, 19–22, 24, 25, 28–30] reported the HDL-C level between PD patients and health controls. There was significant heterogeneity on the synthesized HDL-C level (*I*^2^ = 59%, *P*=0.10); therefore, the random effect model was applied for data synthesis. As shown in Figure 4, meta-analysis indicated that HDL-C level in the PD patients was significantly lower than that of health controls (SMD = −0.14, 95% CI −0.26∼−0.02, *P*=0.02).

**Figure 5:**
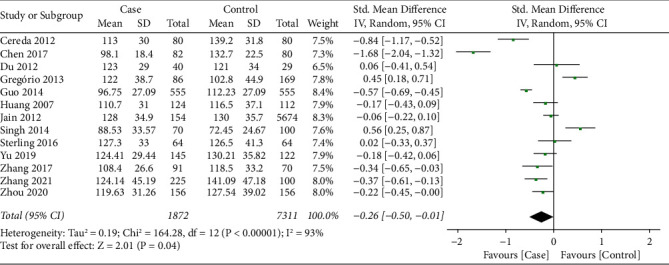
Forest plot for synthesized LDL-C level.

13 studies [16, 18–26, 28–30] reported the LDL-C level between PD patients and health controls. There was significant heterogeneity on the synthesized LDL-C level (*I*^2^ = 93%, *P* < 0.001); therefore, the random effect model was applied for data synthesis. As shown in Figure 5, meta-analysis indicated that LDL-C level in the PD patients was significantly lower than that of health controls (SMD = −0.26, 95% CI −0.50∼−0.01, *P*=0.04).

### 3.4. Subgroup Analysis and Publication Bias Analysis

**Figure 6:**
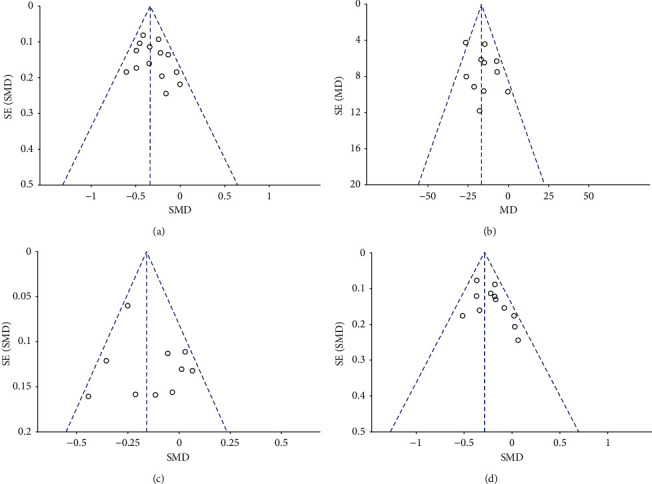
Funnel plots for synthesized outcomes. (a) Funnel plot for synthesized TC level, (b) Funnel plot for synthesized TG level, (c) Funnel plot for synthesized HDL-C level, (d) Funnel plot for synthesized LDL-C level.

The results of TC, HDL-C, and LDL-C have high heterogeneity. To explore the potential sources of high heterogeneity, regression analysis was performed on variables such as age and gender. In the regression analysis, gender had no significant effect on TC (*P*=0.612), HDL-C (*P*=0. 771), and LDL-C (*P*=0.606). Also, age had no significant effect on TC (*P*=0.147), HDL-C (*P*=0.096), and LDL-C (*P*=0.214). In addition, a subgroup analysis based on geographic location shows that the heterogeneity between Asian countries and non-Asian countries is very high. It can be seen that geographic location is not the main source of heterogeneity (Table 2). Sensitivity analysis shows that when one of the studies is omitted, there is no significant change in the combined SMD, indicating that the results are stable. When using Begg's test and funnel plot to measure publication bias, there is no evidence that there is a significant publication bias (Begg's test results, TC: *P*=0.131, TG: *P*=0.465; HDL-C: *P*=0.778, LDL-C: *P*=0.426, Figure 6).

## 4. Discussions

PD is a common neurological disease in middle-aged and elderly people, and its incidence is second only to Alzheimer's disease [33]. PD is mostly insidious and has a chronic progressive course. Patients over 50 years old account for 90% of the total number of PD patients [34, 35]. Studies [36, 37] have reported that the incidence of PD in China is close to 2%, and there are currently more than 2 million PD patients in China. There is currently no complete cure regimen for PD, most patients can continue to attack for a few years, they can also develop rapidly and become disabled, severe muscle rigidity and body stiffness can occur in the late stage of the disease, and eventually bedridden and lose their ability to take care of themselves, which seriously endangers the physical and mental health of the elderly [38, 39]. There is a lack of significant clinical symptoms in the early stage of PD [40]. In addition, there is currently a lack of clinical biomarkers for predicting the risk of PD and the progression of the disease. Therefore, searching for biomarkers related to the risk and severity of PD has become a hot spot in clinical research in recent years. In recent years, blood lipids have become a blood biochemical index that may be related to PD in many clinical studies [41, 42], but the results of the studies are not consistent. The results of our synthesized outcomes have indicated that the TC, TG, HDL-C, and LDL-C level in the PD patients was significantly lower than that of health controls.

The lipids contained in plasma are collectively called blood lipids, which are the general term for neutral fats and lipids. Neutral fats include TG and TC, while lipids include phospholipids, glycolipids, sterols, and steroids, which are essential for basic metabolism of life cells substance [43, 44]. TG is mainly involved in energy metabolism in the human body, while TC is involved in the synthesis of cell plasma membrane, steroid hormones, and bile acids [45]. Previous research results [46–48] have shown that abnormal changes in blood lipid biochemical indicators, such as TC and lipoprotein, play an important role in the formation of arteriosclerosis. Previous studies [49, 50] have found that in PD and other Lewy body diseases, apoE expression occurs in substantia nigra neurons and Lewy bodies, and the expression of apoE receptors is significantly increased. The apoE2 gene has the effect of reducing LDL-C. Low TC in patients with PD may be related to the concentration of serum coenzyme Q10 [51]. TC and coenzyme Q10 have the same biosynthetic pathway, and serum TC is the determinant of serum coenzyme concentration [52]. Coenzyme Q10, as the basis of the electron transporter, is one of the components of the respiratory chain [53]. The reduction of Coenzyme Q10 causes mitochondrial dysfunction, causes degeneration and death of dopaminergic neurons, and ultimately leads to Parkinson's disease [54]. Studies [55, 56] have shown that higher serum TC levels may slow down the clinical progression of PD. The main component of Lewy bodies is *α*-synuclein, and the polymorphism of *α*-synuclein gene rs356219 is related to the pathogenesis of PD. The joint action of alpha-synuclein and lipid oxidation metabolites can cause mitochondrial dysfunction and cause the death of dopaminergic neurons, leading to the occurrence and development of PD [57].

Previous study has found that higher levels of TC, TG, and LDL-C levels are associated with a lower risk of PD [58]. The possible reason is that on the one hand, existing studies have shown that before the occurrence of PD dyskinesia, sympathetic nerve activity has been reduced. Due to the reduction of catecholamine and cortisol production, the blood lipid level may be reduced, and the reduction of blood lipid level may be caused by exercise [59]. On the other hand, lipids have neuroprotective effects. Studies [60, 61] have shown that HDL-C in lipids can directly participate in anti-inflammatory and antioxidant effects. The decrease in HDL-C levels is related to the increased risk of neuronal degeneration. In addition to direct effects, lipids may also affect the occurrence of PD through other ways. The reduction of serum TC and TG levels can reduce the level of serum coenzyme Q10. Coenzyme Q10 has a powerful neuroprotective effect and the function of reducing oxidative stress, which can delay the consumption of dopamine [62]. Therefore, TG can play a neuroprotective effect through coenzyme Q10, thereby affecting the occurrence of PD [63]. In addition, the pathology and pathogenesis of PD involve iron deposition and accumulation, and the substantia nigra may be the main area of iron deposition [64]. Studies [65, 66] have found that lipids and lipoproteins can bind to ferrous iron in dopamine neurons in the substantia nigra, and then store them in lysosomes through autophagy to prevent iron-induced oxidative stress and neurodegeneration. Therefore, the reduction in blood lipid levels may occur before the occurrence of PD, which has a certain predictive effect on the occurrence of PD, and maintaining a certain level of TC, TG, HDL-C, and LDL-C may prevent the occurrence and development of PD.

Cholesterol is an important component of the cell membrane, has a dual role in structure and function, and can regulate a variety of signal pathways and cell functions [67]. Abnormal cholesterol levels may be related to neurodegenerative diseases and may be a potential risk factor for the onset of Parkinson's disease [38]. Higher serum TC may be related to the slow clinical progression of PD. Studies [68, 69] have reported that LDL-C is associated with the risk of Parkinson's disease, and elevated LDL-C levels may also delay the progression of PD [42]. The link between hypolipidemia and PD is complex, and it is not fully understood at present. To explain the relationship between them, further research is needed, such as expanding the research sample size and perfecting related basic research, in order to clarify the relationship between blood lipid levels and PD dyskinesia and cognitive impairment, to provide insights to the PD prevention, treatment, and nursing strategies.

## 5. Conclusions

In conclusion, the results of this study show that hypolipidemia including lower TC, TG, HDL-C, and LDL-C may be related to the pathogenesis and development of PD. Statins are commonly used clinically lipid-lowering drugs, but whether statins are currently beneficial to patients with PD remains to be further studied. It is worth noting that there is still a lack of comparisons of different age groups in terms of nutritional status and different populations, and the associations between blood lipids and development of PD still need further investigations in the future.

## Figures and Tables

**Table 1:** The characteristics of included studies.

	Country	Sample	Female (%)	Age (y)	Duration of diseases (y)	TC (mg/dl)	TG (mg/dl)	LDL-C (mg/dl)	HDL-C (mg/dl)	NOS
		Case/control	Case/control	Case/control	Case/control	Case/control	Case/control	Case/control	Case/control	
Cereda et al. [16]	Italy	80/80	47.5/47.5	61.5 ± 10.2/58.6 ± 9.1	NA	186.1 ± 30.9/220.8 ± 34.8	102.1 ± 38.1/128.3 ± 60.8	113.0 ± 30.0/139.2 ± 31.8	52.9 ± 15.3/55.9 ± 12.7	7
Chen 2017	China	82/80	43.9/41.9	70.4 ± 10.6/74.9 ± 9.7	NA	180.4 ± 42.1/237.4 ± 45.5	108.3 ± 25.6/123.0 ± 30.7	98.1 ± 18.4/132.7 ± 22.5	49.9 ± 12.8/56.3 ± 10.3	8
Dong et al. [17]	China	76/61	40.8/52.5	69.3 ± 8.99/67.9 ± 5.74	NA	164.48 ± 32.51/162.15 ± 35.22	110.75 ± 67.34/128.47 ± 69.99	NA	NA	8
Du et al. [18]	USA	40/29	42.5/58.6	60.7 ± 8.3/59.6 ± 6.7	4.2 ± 4.7/0	195 ± 36/201 ± 39	NA	123 ± 29/121 ± 34	NA	7
Gregório et al. [19]	Brazil	86/169	NA	NA	NA	202.5 ± 45.7/186.8 ± 51.1	115.5 ± 66.7/136.9 ± 73.5	122.0 ± 38.7/102.8 ± 44.9	58.0 ± 13.2/56.8 ± 20.9	8
Guo et al. [31]	China	555/555	42.9/42.9	62.2 ± 12.2/62.4 ± 12.7	NA	174.15 ± 34.83/193.5 ± 34.83	106.32 ± 70.88/132.9 ± 70.88	96.75 ± 27.09/112.23 ± 27.09	58.05 ± 15.48/61.92 ± 15.48	6
Huang et al. [22]	USA	124/112	44.4/55.4	67.9 ± 10.4/65.7 ± 10.9	4.2 ± 6.1	191.6 ± 38.4/200.7 ± 43.8	NA	110.7 ± 31.0/116.5 ± 37.1	54.6 ± 17.3/54.4 ± 17.0	6
Jain et al. [23]	USA	154/5674	NA	NA	NA	206 ± 40.6/211 ± 39.2	NA	128 ± 34.9/130 ± 35.7	NA	8
Singh et al. [25]	India	70/100	NA	NA	NA	168.17 ± 41.0/124.12 ± 31.13	159.32 ± 67.97/159.63 ± 49.92	88.53 ± 33.57/72.45 ± 24.67	46.76 ± 10.24/47.19 ± 9.10	8
Sterling et al. [26]	USA	64/64	40.6/50	62.7 ± 7.9/61.3 ± 6.8	4.4 ± 4.4/0	NA	NA	127.3 ± 33.0/126.5 ± 41.3	NA	7
Vikdahl et al. [27]	Sweden	84/336	45.2/45.2	NA	NA	229.88 ± 44.12/232.21 ± 46.05	117.84 ± 42.53/134.67/74.42	NA	NA	7
Yu et al. [32]	China	145/122	51.0/54.92	67.99 ± 8.88/64.27 ± 9.52	4.5 ± 4.07/0	201.29 ± 35.02/227.73 ± 40.56	131.22 ± 60.45/138.36 ± 61.98	124.41 ± 29.44/130.21 ± 35.82	48.16 ± 9.94/47.09 ± 10.16	8
Zhang et al. [28]	USA	91/70	46.1/45.7	65.8 ± 11.2/63.5 ± 11.6	NA	183.0 ± 33.5/195.2 ± 37.1	108.0 ± 51.0/123.4 ± 66.6	108.4 ± 26.6/118.5 ± 33.2	50.3 ± 15.6/52.1 ± 15.7	6
Song et al. [33]	China	225/100	38.7/47.00	61.26 ± 10.73/59.65 ± 7.32	NA	193.48 ± 40.35/225.12 ± 39.14	124.04 ± 41.93/139.17 ± 58.25	124.14 ± 45.19/141.09 ± 47.18	46.29 ± 18.43/53.21 ± 21.45	9
Zhou 2020[45]	China	156/156	39.74/42.31	68.61 ± 8.36/66.93 ± 7.84	3.48 ± 3.1/0	199.03 ± 36.24/212.05 ± 39.88	139.15 ± 62.07/146.61 ± 48.44	119.63 ± 31.26/127.54 ± 39.02	54.12 ± 18.06/54.95 ± 12.17	8

**Table 2:** Subgroup analysis stratified by geographic location.

Items	Subgroup	Number of studies	SMD (95%CI)	*I* ^2^	*P* value
TC	Asian countries	7	−0.26 (−1.82, −0.01)	89	0.03
	Non-Asian countries	7	−0.22 (−0.79, −0.05)	86	0.04

TG	Asian countries	6	−0.16 (−1.04, −0.02)	79	0.04
	Non-Asian countries	5	−0.15 (−1.12, −0.08)	84	0.03

LDL-C	Asian countries	6	−0.09 (−0.53, −0.01)	90	0.01
	Non-Asian countries	7	−0.15 (−1.22, −0.07)	82	0.02

HDL-C	Asian countries	6	−0.24 (−1.95, −0.11)	77	0.01
	Non-Asian countries	4	−0.18 (−1.04, −0.03)	79	0.04

## Data Availability

All the data generated or analyzed during this study are included in this published article.

## Supplementary Materials

Supplementary Materials Supplementary file 1: search strategy for PubMed.
