# Supplementary material for: Lower Blood Lipid Level Is Associated with the Occurrence of Parkinson's Disease: A Meta-Analysis and Systematic Review
Source: Int J Clin Pract. 2022 Jun 9;2022:9773038. doi: 10.1155/2022/9773038 (PMC9203242; doi:10.1155/2022/9773038)
Supplement: Supplementary Materials — Supplementary file 1: search strategy for PubMed. [file 9773038.f1.pdf]

Supplementary file 1 Search strategy for Pubmed

1:
("Parkinson disease "[Mesh] OR " Parkinson"[Mesh] OR "PD"[Mesh]) AND (“serum lipid” OR “blood lipid” OR “dyslipidemia” OR “cholesterol” OR “triglyceride” OR “low density lipoprotein cholesterol” OR “LDL-C "OR "high density lipoprotein cholesterol" OR "HDL-C" OR " cholesterol "[Mesh])
